# Constipation with bloating: a clinical approach to evaluation and management

**DOI:** 10.1080/07853890.2026.2684172

**Published:** 2026-06-19

**Authors:** David J. Cangemi, Adam P. Laitman, Orit Faiman, Bianca W. Chang

**Affiliations:** ^a^ Division of Gastroenterology & Hepatology, Mayo Clinic, Jacksonville, FL, USA; ^b^ Department of Medical Affairs, Salix Pharmaceuticals, Inc, Bridgewater, NJ, USA; ^c^ Karsh Division of Gastroenterology and Hepatology, Cedars-Sinai Medical Center, Los Angeles, CA, USA

**Keywords:** Bloating, chronic idiopathic constipation, IBS-C, intestinal methanogen overgrowth, irritable bowel syndrome, pelvic floor dysfunction

## Abstract

**Introduction:**

Constipation and bloating are common bothersome gastrointestinal (GI) symptoms that frequently occur concurrently. It is important to recognize the underlying condition(s) and tailor therapeutic interventions appropriately. This review discusses the evaluation and management of patients with constipation who also have bloating as a predominant symptom.

**Discussion:**

Key areas reviewed are potential etiologies for constipation with bloating and clinical evaluation, including differentiation between common underlying causes such as chronic idiopathic constipation (CIC) and irritable bowel syndrome with constipation (IBS-C). Management approaches discussed include dietary modification, biofeedback, and pharmacologic therapy, including over-the-counter treatments, plecanatide, linaclotide, lubiprostone, tenapanor, neuromodulators, and antibiotics.

**Conclusions:**

Constipation and bloating are common GI symptoms; however, bloating is a relatively nonspecific symptom with a complex pathophysiology. While specifically treating constipation may also improve bloating, this strategy does not improve bloating in all patients. There is a need for additional research to further understand the physiology of bloating. In an era when personalized medicine is becoming increasingly emphasized, understanding the pathophysiology of bloating for an individual patient, be it alongside constipation or other GI and non-GI symptoms, will be paramount to improve treatment outcomes.

## Key Messages

- Patients presenting with constipation, with bloating as a predominant symptom, should be evaluated for underlying conditions, including irritable bowel syndrome with constipation (IBS-C), chronic idiopathic constipation (CIC), pelvic floor dysfunction, and microbial overgrowth (e.g. intestinal methanogen overgrowth), among other causes
- If food intolerances are suspected, a trial of dietary restriction, such as a low-fermentable oligosaccharides, disaccharides, monosaccharides, and polyols diet, can be suggested
- Improvement in bloating has been demonstrated in large, randomized trials of several gastrointestinal-targeted pharmacologic therapies (e.g. secretagogues) in patients with IBS-C or CIC
- Pelvic floor physical therapy with biofeedback should be considered for patients with dyssynergic defecation

## Introduction

Constipation and bloating are common bothersome gastrointestinal (GI) symptoms [1,2]. The estimated prevalence of constipation has ranged from 6% to 9% in Asian countries to 15% to 17% in Europe and North America [3]. The estimated prevalence of bloating has ranged from approximately 11% in East Asian countries to approximately 20% in European, Latin American, Middle Eastern, and North American countries [4]. Sociocultural factors likely contribute to differences in patient perception of symptoms and reported prevalence rates [3,5].

Constipation and bloating frequently present concurrently [6,7]. In a global survey of 51,425 people with nonorganic causes of GI symptoms, approximately one-third of individuals reporting constipation with ≥30% of their bowel movements also reported bloating at least once per week [4]. Rates of bloating were higher among individuals with irritable bowel syndrome with constipation (IBS-C) or chronic idiopathic constipation (CIC), with 71% and 36% of these populations, respectively, reporting bloating at least once weekly [4]. In another global survey (*N* = 20,099), among 10,425 (51.9%) individuals who reported having constipation in the previous 6 months, 65% reported also having bloating/distention [7]. Of note, a study in Italy of patients with IBS-C (*n* = 369) or CIC (*n* = 1834) identified substantially higher bloating prevalence rates of 96% and 91%, respectively, based on a 2-week recall period [1]. Although patients may often experience bloating and abdominal distention concurrently, these are distinct phenomena. Distension refers to an objective measurable increase in abdominal girth, whereas bloating refers to a subjective sensation of gassiness or fullness/pressure within the abdomen and does not include visible distension [2]. The underlying pathophysiology of bloating is complex and diverse, as will be discussed latter in the manuscript, whereas abdominal distension may result from an increased volume of gas or stool within the intestines. Abdominophrenic dyssynergia, in which the diaphragm inappropriately contracts and the anterior wall muscles inappropriately relax in response to intestinal gas load, leading to distension, remains an underrecognized cause of abdominal distension, particularly in disorders of gut-brain interaction [8]. Data have shown that both constipation and bloating impair patient health-related quality of life (QoL) [1,9]. Constipation is associated with pronounced detriments to general health, social functioning, and mental health, leading to impairments in health-related QoL that are comparable to those associated with chronic diseases, such as diabetes and osteoarthritis [10]. Chronic constipation has been associated with poor QoL and impaired general well-being [11–13], with a strong negative correlation between symptom severity and QoL [12]. Individuals with CIC experience markedly impaired QoL compared with individuals without constipation [10,14]; likewise, individuals with IBS-C report greater impairments in health-related QoL, work productivity, and activity compared with those without IBS-C [15]. Notably, patients with bloating have impaired QoL, independent of CIC or IBS-C [1]. In a US survey of patients with IBS-C (*n* = 328) or CIC (*n* = 552), bloating was reported as a ‘very/extremely bothersome’ symptom in 56% of patients with IBS-C and 46% of patients with CIC (*p* < 0.03 between the 2 groups) [16]. Additionally, the frequency of bloating was higher in patients with IBS-C vs CIC (2.8 vs 1.4 mean days/week, *p* < 0.0001). A systematic literature review of studies of CIC that used validated or disease-specific QoL measures, including the Patient Assessment of Constipation Quality of Life questionnaire, concluded that increased intensity of symptoms of constipation, including bloating, correlated with impaired QoL [17].

Given that constipation and bloating are common symptoms that can occur with a range of medical conditions, it is crucial to recognize the underlying condition(s) and tailor therapeutic interventions appropriately. The objective of this narrative review is to highlight key considerations in the evaluation and management of adults with constipation who also have the subjective sensation of bloating as a clinically predominant (i.e. bothersome) symptom.

## Methods

PubMed was searched for English-language publications on constipation with bloating during a 10-year period (January 2015 through August 2025). The search string applied was ‘constipation’ AND ‘bloating’ AND (‘irritable bowel syndrome’ OR ‘chronic idiopathic constipation’ OR ‘functional constipation’). For pharmacologic treatments indicated for IBS-C or CIC, evidence was prioritized from randomized controlled trials (RCTs), pooled analyses of RCTs, and meta-analyses. When RCT data were not consistently available (e.g. nonpharmacologic therapies), other clinical trials and meta-analyses were also reviewed. Study appraisal prioritized publications on relevant therapies that reported both constipation and bloating as individual outcomes. Reference lists of all included articles were manually screened to identify additional relevant publications not captured in the primary search. Given the narrative design, a formal risk-of-bias assessment was not performed.

## Etiology of constipation with bloating symptoms

**Figure 1.**
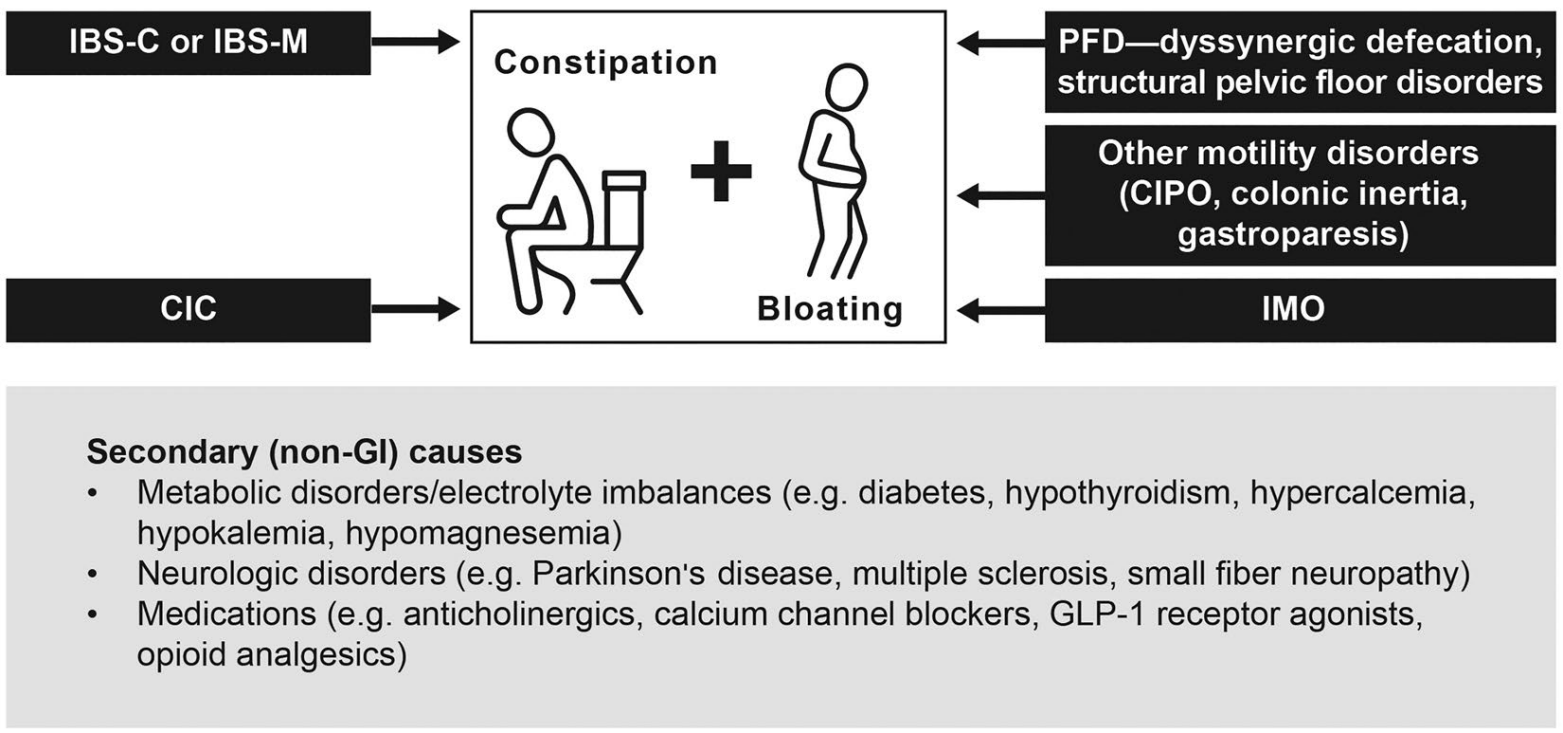
Etiology of constipation with bloating [6,18–29]. CIC: chronic idiopathic constipation; CIPO: chronic intestinal pseudo-obstruction; GI: gastrointestinal; GLP-1: glucagon-like peptide-1; IBS-C: irritable bowel syndrome with constipation; IBS-M: irritable bowel syndrome mixed type; IMO: intestinal methanogen overgrowth; PFD: pelvic floor dysfunction.

Several GI disorders should be considered in an adult experiencing constipation with bloating, including CIC, IBS (predominant constipation [IBS-C] or mixed type [IBS-M]), pelvic floor dysfunction (PFD; e.g. dyssynergic defecation and structural pelvic floor abnormalities), and intestinal microbial overgrowth, specifically intestinal methanogen overgrowth (IMO). Additionally, though bloating can occur in the setting of impaired GI motility, motility disorders, such as gastroparesis and chronic intestinal pseudo-obstruction, are relatively uncommon (Figure 1) [6,18–30]. Non-GI, secondary causes of constipation should also be considered [20]. Constipation may be secondary to or exacerbated by metabolic disorders (e.g. diabetic neuropathy and hypothyroidism), electrolyte imbalances/abnormalities (e.g. hypercalcemia, hypokalemia, and hypomagnesemia), endometriosis, and neurologic disorders (e.g. Parkinson’s disease and small fiber neuropathy), among other causes [20,26–29,31]. Inquiry about prescription and over-the-counter (OTC) product use is important because many commonly administered medications, such as anticholinergics, calcium-containing antacids, opioid analgesics, and dietary supplements (e.g. iron, calcium) may cause constipation [20]. This article focuses on potential GI-related causes; readers seeking additional information on secondary etiologies should consider reviewing these cited references [32–34]. Celiac disease (i.e. autoimmunity characterized by abnormal reaction to gluten in genetically susceptible individuals), nonceliac gluten sensitivity (i.e. reaction to gluten that is not mediated by allergy or autoimmunity), and food intolerance are potential underlying contributors to bloating; however, these conditions more commonly present with diarrhea than constipation [19,35].

## Approach to evaluation and management of constipation with bloating

**Figure 2.**
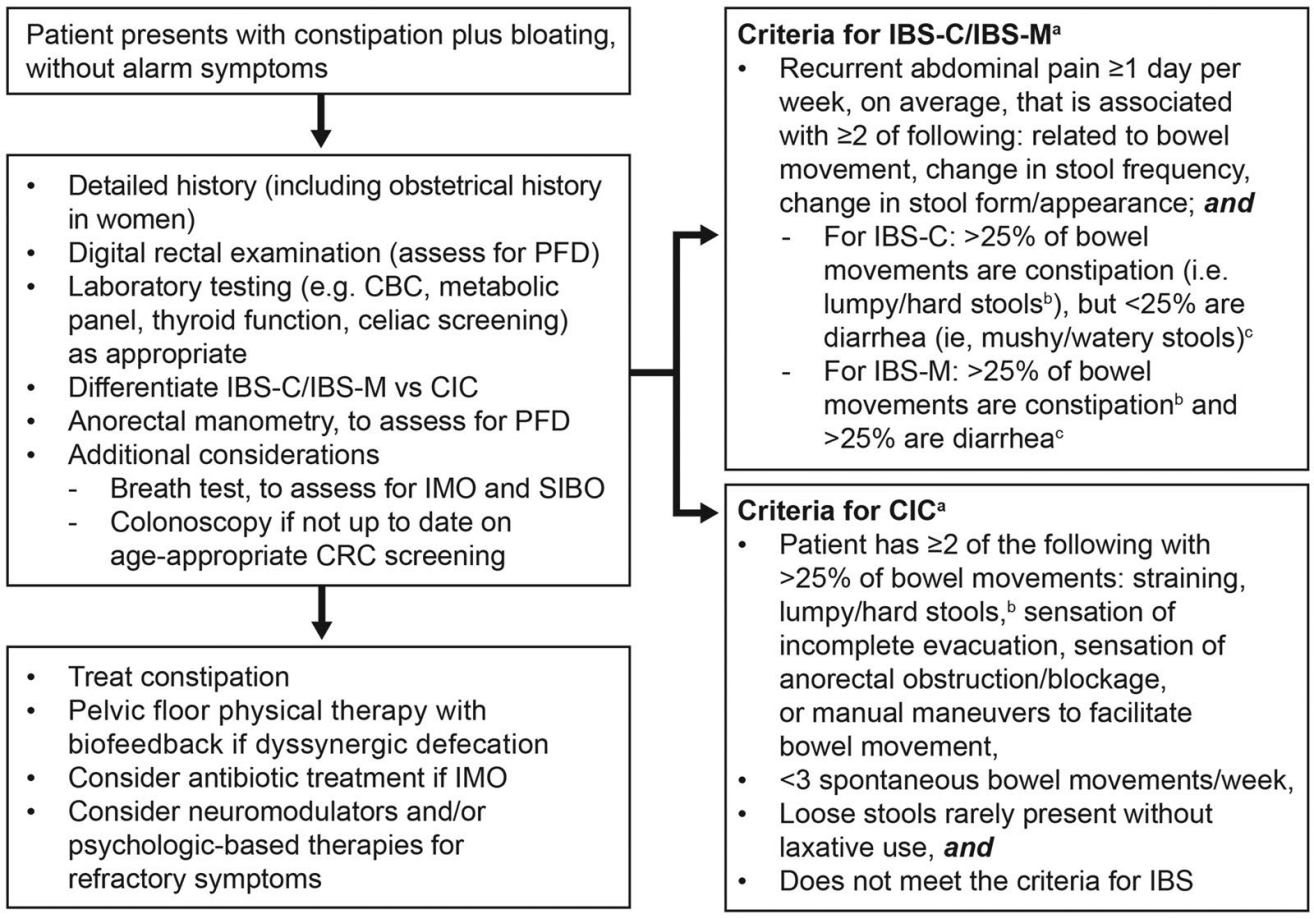
Clinical approach to constipation with bloating [2,19,20,36–39]. ^a^Time frame for criteria is within the previous 3 months, with onset ≥6 months before diagnosis. ^b^Bristol Stool Form Scale Types 1 or 2 [37]. ^c^Bristol Stool Form Scale Types 6 or 7 [37]. CBC: complete blood count; CIC: chronic idiopathic constipation; CRC: colorectal cancer; IBS-C: irritable bowel syndrome with constipation; IBS-M: irritable bowel syndrome mixed type; IMO: intestinal methanogen overgrowth; PFD: pelvic floor dysfunction; SIBO: small intestinal bacterial overgrowth.

An approach to evaluating and managing a patient presenting with constipation with bloating, including medical history, basic laboratory testing, physical examination, and primary diagnostic considerations, is outlined in Figure 2 [2,19,20,36–39].

### Evaluation

#### History and physical

In a patient presenting with constipation and bloating, an initial evaluation should include identification of alarm symptoms (e.g. iron-deficiency anemia of unknown etiology, GI bleeding, vomiting, unintentional loss of >10% of body weight, and a family history of GI cancer or inflammatory bowel disease), as the aforementioned factors should influence testing [2,19]. Diagnostic workup should begin with a detailed history. In women, obstetric history is crucial, given that more than half of women may have PFD within the decade following childbirth [40]. Vaginal delivery, in particular, is associated with an increased risk of PFD [41], potentially due to pudendal nerve damage and persistent functional impairment in the pelvic floor sphincter musculature [42].

A physical, including a digital rectal examination (DRE) is essential, and a comprehensive 10-step approach to a DRE in GI practice has been described previously [43]. A DRE may reveal stricture, spasm, tenderness, mass, blood, and/or presence of stool [21] and can also help identify pertinent structural factors (e.g. rectocele in the anterior wall) [44]. During the DRE, for GI-related assessment, the patient should lie on their left side [43], and they should push and bear down as though having a bowel movement, so the examiner can assess for appropriate anal sphincter and puborectalis muscle relaxation and perineal descent, as well as any obvious extrinsic compression. Simultaneously, the examiner should place their hand on the patient’s abdomen to assess for a strong abdominal push effort. If the examiner does not detect appropriate relaxation of the anal sphincter muscles with the bear-down maneuver, an evacuation disorder should be suspected. To further assess for PFD, the provider may consider testing with high-resolution anorectal manometry (ARM), ideally with balloon expulsion testing. Notably, ARM testing is not widely available, but it is commonly offered at referral practices and academic institutions. If ARM testing is not available, a careful DRE should be performed, as described above, to assess for PFD.

Data suggest that ARM detects the health status (i.e. presence/absence of dyssynergic defection) in only approximately 60% of patients [45,46]. When resources are available, defecography can be considered a first-line diagnostic test to determine whether structural abnormalities contribute to chronic constipation [45]. Additional diagnostic modalities that may be useful in evaluating patients with constipation and bloating include wireless motility capsules (e.g. ingestible gas-sensing capsule) and radionucleotide scintigraphy, which assesses GI transit and motility [47–49].

Constipation and bloating are both characteristic bowel symptoms of PFD [50]. Structural pelvic floor abnormalities (e.g. rectocele, enterocele, and cystocele) can cause obstructed defecation [51–53], which, in turn, may contribute to bloating. Clinically relevant rectoceles (>2 cm) are common in women with constipation and can impair rectal emptying, particularly when large in size [30]. Even in patients with constipation who do not exhibit overt outlet obstruction, it is important to consider dyssynergic defecation when bloating is a predominant symptom. Dyssynergic defecation is a type of PFD characterized by impaired coordination between the pelvic floor muscles, anal sphincter, and abdominal wall during attempted defecation [20]. Symptoms most predictive of dyssynergic defecation include a sense of incomplete evacuation or blockage, excessive straining, and the use of digital maneuvers [54]. Abdominal bloating is also common in this population and is reported as occurring ‘often’ to ‘always’ by almost three-fourths of patients [55]. Additionally, patients may have urinary or sexual symptoms (i.e. dyspareunia) that are associated with other conditions under the umbrella term of PFD [21].

Of interest, a 2024 study in patients with chronic constipation identified a minimally invasive and reliable approach for determining which individuals may benefit from early referral to biofeedback. Two findings were strongly associated with dyssynergic defection: (1) failed anal relaxation during simulated defection on DRE, particularly when abdominal palpation is added during straining, and (2) response of ‘anal muscles’ when asked, ‘What muscles do you mainly use when you push to defecate?’ [56].

After obtaining a detailed medical history and conducting a comprehensive physical exam, a basic laboratory workup could be considered, especially in patients who have not had recent testing. This may include a complete blood count, a comprehensive metabolic panel, thyroid function tests (i.e. thyroid-stimulating hormone and free thyroxine levels), and celiac screening [57] (i.e. tissue transglutaminase immunoglobulin A and total serum immunoglobulin A levels). These evaluations may be performed in a primary care or specialty setting as part of the initial diagnostic assessment. It is important, however, to tailor laboratory testing to the individual, as not all patients require the same level of investigation.

##### IBS and CIC

Differentiation among IBS-C, IBS-M, and CIC as potential underlying causes of constipation with bloating is important (see Figure 2 for diagnostic criteria). IBS-C and CIC have multiple overlapping symptoms; however, a key point of distinction is that patients with IBS, by definition, have recurrent abdominal pain, whereas pain is not fundamental to the diagnosis of CIC [36]. When pain is present in CIC, it is relatively less frequent and less intense. Patients with IBS-C also report a higher frequency and intensity of bloating compared with individuals with CIC [58].

#### Other considerations

Colonoscopy may be advised for patients who are not current with age-appropriate screening for colorectal cancer, but it would not be recommended for evaluation of bloating and constipation symptoms, particularly without alarm features, in a young, otherwise healthy individual. In patients in whom dyssynergic defecation is ruled out, a colonic transit study (e.g. Sitz marker test) may be considered, if available, to assess for slow-transit constipation or colonic inertia; however, this is a relatively uncommon condition [20,24]. In patients with clear risk factors for intestinal microbial overgrowth (e.g. conditions associated with abnormal small intestinal motility or anatomic abnormalities such as small bowel diverticula or procedures including surgeries of the ileocecal valve and Roux-en-Y gastric bypass) [59] or severe symptoms, breath testing (utilizing glucose or lactulose as substrate) may be warranted [19,59]. While both IMO and small intestinal bacterial overgrowth (SIBO) are associated with bloating, IMO has a much stronger association with constipation than SIBO [18,59–61]. *Methanobrevibacter smithii* is the predominant methanogen in the human GI tract, and in patients with IMO, *M. smithii* produces excess methane, which causes bloating, distention, and worsening constipation [18,60,62–64].

Breath testing is a noninvasive diagnostic tool that may be considered in select patients with supportive clinical features and a high symptom burden, although its use is supported by limited evidence. Because there is a lack of consensus on optimal cutoff times, diagnostic thresholds, and substrate dosing, routine breath testing may not be warranted for the evaluation of bloating/distention, unless a patient has clear risk factors or severe symptoms [2,19]. Glucose and lactulose are the primary substrates used for breath testing; glucose, due to its rapid absorption, is associated with fewer false-positive results, although false positives can still occur [65–67]. Exhaled breath hydrogen and methane serve as biomarkers of metabolically active gut microbes – a hydrogen-predominant result suggests SIBO and a methane-predominant result indicates IMO [65,66]. In patients with IBS-C, bloating may be caused by IMO, and the North American consensus document recommends a diagnostic methane threshold of ≥10 parts per million at any time point during testing [61]. A 2022 study showed high sensitivity and specificity for diagnosing IMO using a methane threshold of ≥10 parts per million [68]. However, the optimal criterion for defining excessive methane production is still being debated, and further validation studies are warranted [61].

Key limitations of breath testing include its indirect measurement of microbial overgrowth, reliance on patient adherence to pretesting preparation protocols (e.g. avoidance of fermentable foods for 24 h and avoidance of exercise and smoking on test day), and impaired substrate transit in those with comorbid conditions, such as gastroparesis [66]. For SIBO, variable orocecal transit time can further confound interpretation [65]. Clinically, IMO can only be diagnosed with breath testing, whereas SIBO may also be diagnosed by small bowel aspirate. However, aspirate culture, often described as the gold standard, is rarely performed due to its invasiveness, susceptibility to contamination from oropharyngeal or gastric flora [66,69], and the fact that >60% to 70% of gut microbes cannot be identified or cultured using conventional techniques [69,70]. The lack of a validated gold standard test for diagnosing SIBO has been identified as a key challenge in breath testing [61]. Notably, diagnostic criteria for methane breath tests have never been validated [71]. Several recent publications further delve into the role of breath testing, including controversies [66,67,71].

For patients with a high pretest probability of celiac disease (e.g. first-degree relative with the condition, presence of associated autoimmune disorders), serologic testing using tissue transglutaminase and immunoglobulin A is warranted [2,72]. Although celiac disease is typically associated with diarrhea rather than constipation, health care providers should consider serologic testing for celiac disease if a patient with constipation continues to have persistent bloating after initiation of treatment (see the Management section below) [2,36].

### Management

Several treatment options are available to manage symptoms in patients with constipation and bloating. Interventions include dietary modification, pharmacologic treatments, and psychotherapeutic approaches (e.g. cognitive-behavioral therapy [CBT] or gut-directed hypnotherapy for IBS; biofeedback for dyssynergic defecation) [2,39,73–75]. It has been suggested that certain OTC products for constipation, including psyllium fiber and the laxative polyethylene glycol, may worsen bloating [76,77]; however, a minimal or neutral impact on bloating has also been described [78–80]. That said, soluble fiber and polyethylene glycol have favorable safety profiles and may be considered reasonable first-line treatments for patients with constipation and bloating.

#### Dietary modification

If food intolerance is suspected, a brief (e.g. 2-week) trial of dietary restriction can be employed to assess for resolution of symptoms [19]. However, dietary restriction should generally be avoided in individuals with a history of an eating disorder (e.g. avoidant restrictive food intake disorder) and approached with caution in those at increased risk for disordered eating [81]. The low-fermentable oligosaccharides, disaccharides, monosaccharides, and polyols (FODMAP) diet has been shown to improve bloating and distention in patients with IBS as well as non-IBS etiologies [2,82–84]. If symptom resolution does not occur during the trial, the diet should be discontinued and other treatment options considered [81]. If symptoms improve, the global restriction of FODMAP intake should be continued for a total of 4 to 8 weeks before transitioning to the gradual reintroduction phase. During reintroduction, previously eliminated foods are methodically added back while monitoring for recurrence of symptoms, with the goal of avoiding nutritional deficiencies [85].

Practical challenges of the low-FODMAP diet include patients finding it too complicated to follow, as well as it being expensive and unpalatable [86]. In a study of individuals with IBS (*n* = 131) or inflammatory bowel disease (*n* = 49), approximately one-quarter (26%) of the participants with IBS discontinued the diet before completing the initial dietary period, and fewer than half (47%) remained on it at follow-up (median, 15 months) [86]. Long-term maintenance is particularly difficult, given the extent of food exclusions and the protracted nature of the weekly food reintroduction process [87]. An umbrella review of 16 low-FODMAP diet meta-analyses of approximately 10,000 adults with IBS reported reduced overall symptom severity and improved QoL [88]. However, no statistically significant effect was demonstrated on key symptoms, including bloating and bowel movement frequency, and the majority of data were from short-term trials with variable adherence reporting.

Because reducing consumption of high FODMAP fruits and vegetables can decrease fiber intake, some patients may be at risk of constipation unless the restricted items are replaced with lightly fermentable, high-fiber alternatives (e.g. oat or rice bran) [85]. Notably, the 2021 guideline from the American College of Gastroenterology recommends that soluble, but not insoluble, fiber be used to treat global IBS symptoms [89]. This recommendation may be particularly helpful for patients with IBS-C, as fiber may improve stool frequency and viscosity. However, the data supporting this rationale in IBS-C is considered weak [89].

Collaboration with a skilled, qualified dietician is essential to guide patients through the restriction and reintroduction phases of the low FODMAP diet, ensure adequate nutrition, and support long-term diet personalization. While the role of gluten in bloating is less established than that of FODMAPs [2], a gluten-free diet should be instituted, under the guidance of a dietician, in patients with confirmed celiac disease. In cases in which nonceliac gluten sensitivity is suspected, a gluten-free diet can be implemented for at least 2 weeks to assess symptom improvement, followed by a gluten challenge for diagnostic confirmation [19,35].

In 2025, the British Dietetic Association published the first evidence-based guidelines on the dietary management of chronic constipation in adults [78]. Among the dietary options recommended as effective for constipation, psyllium supplements, select probiotic strains, and kiwifruit did not demonstrate an effect on bloating, whereas administration of magnesium oxide supplements was associated with reduced bloating severity in patients with constipation [78,90]. The guidelines did not recommend whole diet approaches, such as a high-fiber diet, because of insufficient evidence supporting their efficacy [78].

##### Pharmacologic treatments indicated for the management of IBS-C and CIC

Several medications indicated for the treatment of IBS-C and/or CIC have had findings reported for the treatment of bloating, independent of constipation symptom management (Table 1) [74,91–102]. Of note, a meta-analysis (*n* = 13 trials; 10,091 patients) affirmed the effectiveness of prosecretory agents as an overall class for the treatment of bloating in patients with IBS-C [73]. The secretagogue plecanatide, a guanylate cyclase-C agonist indicated for the treatment of IBS-C and CIC in adults, was evaluated in two identically designed, phase 3, randomized, double-blind, placebo-controlled trials in patients with IBS-C [74]. In a pooled analysis, plecanatide 3 mg once daily for 12 weeks significantly improved bloating from baseline compared with placebo as early as Week 1, with sustained significant improvement across the 12-week treatment period (*p* < 0.001) [74]. Plecanatide was also evaluated in two identically designed, phase 3, randomized, double-blind, placebo-controlled trials in patients with CIC [95,96]. In both trials, plecanatide 3 mg once daily for 12 weeks significantly improved bloating versus placebo (*p* < 0.01) [95,96]. A set of subgroup analyses from these trials, of individuals meeting criteria for severe constipation, reported significant reductions in bloating with plecanatide 3 mg versus placebo in both IBS-C (*p* = 0.001) and CIC (*p* < 0.001) [100].

**Table 1.** Impact on bloating: pharmacologic agents indicated for IBS-C and/or CIC.

Pharmacologic agent	Mechanism	Population(s)	Impact on bloating^a^
Plecanatide [74,95,96,100]	Guanylate cyclase-C agonist	IBS-C; CIC	Significant improvement in bloating in both IBS-C and CIC, including individuals with severe constipation at baseline
Linaclotide [91,92,97,98]	Guanylate cyclase-C agonist	IBS-C; CIC	Significant improvement in bloating in both IBS-C and CIC, including individuals with moderate to severe bloating at baseline
Lubiprostone [93,99]	Chloride channel activator	IBS-C; CIC	Significant improvement in bloating in CIC Improvement in IBS-C not consistently significant
Tenapanor [94,102]	Sodium/hydrogen exchanger isoform 3 inhibitor	IBS-C	Significant (higher) bloating responder rate
Prucalopride [101]	Prokinetic selective serotonin-4 receptor agonist	CIC^b^	Improvement in bloating severity (*p* values not available)

^a^”Significant” refers to comparisons of active treatment with placebo with *p* values < 0.05 in clinical trials or pooled analyses.^b^Individuals with moderate to very severe bloating. CIC: chronic idiopathic constipation; IBS-C: irritable bowel syndrome with constipation.

Another guanylate cyclase-C agonist, linaclotide, which is indicated for the treatment of IBS-C and CIC in adults and functional constipation in patients aged 6–17 years, was evaluated in two phase 3, double-blind, placebo-controlled IBS-C trials – a 12-week study and a 26-week study [91,92]. Both studies reported significant improvement from baseline in bloating with linaclotide 290 µg once daily compared with placebo (*p* < 0.0001) [91,92]. Additionally, two randomized, double-blind, placebo-controlled CIC trials reported that linaclotide 145 µg significantly improved bloating versus placebo (*p* < 0.01) [97]. In a randomized, double-blind, 12-week, placebo-controlled CIC trial in individuals with moderate to severe bloating, a significantly greater improvement from baseline in bloating was observed for linaclotide 145 µg versus placebo (*p* < 0.001) [98].

The chloride channel activator lubiprostone, indicated for the treatment of women with IBS-C, adults with CIC, and adults with opioid-induced constipation and chronic noncancer pain, was evaluated in two phase 3, randomized, double-blind, placebo-controlled IBS-C trials [93]. Significant improvement from baseline in bloating was noted with lubiprostone 8 µg twice daily for 12 weeks compared with placebo at Month 2 (*p* = 0.04) but not at Months 1 or 3 (pooled analysis). A meta-analysis (*n* = 9 trials) of 2309 patients with IBS-C or CIC treated with lubiprostone (dose range, 24–72 µg daily*)* reported that improvement in bloating was significantly greater with lubiprostone versus placebo (both conditions pooled; *p* < 0.001) [99]. However, when analyzed by condition, improvement in bloating with lubiprostone versus placebo was significant in patients with CIC (*n* = 900; *p* = 0.004) but not in those with IBS-C (*n* = 1409; *p* = 0.05) [99].

Tenapanor, the sodium/hydrogen exchanger isoform 3 inhibitor, is indicated for the treatment of IBS-C in adults and was evaluated in phase 3, double-blind, placebo-controlled trials lasting 12 and 26 weeks [94,102]. The 12-week study (*n* = 629) examined the impact of tenapanor 50 mg twice daily on bloating and identified that the bloating responder rate (≥30% improvement from baseline in average weekly score for ≥6 of 12 weeks) was significantly higher for tenapanor compared with placebo (*p* = 0.014), whereas the 26-week study did not evaluate tenapanor on the individual symptoms of bloating.

The prokinetic selective serotonin-4 receptor agonist prucalopride is indicated for the treatment of CIC in adults and was evaluated in a post hoc analysis of randomized, double-blind, placebo-controlled CIC trials (five phase 3 trials; one phase 4 trial) in 1931 patients with moderate to very severe bloating at baseline [101]. An approximately 20% greater improvement from baseline in bloating scores was observed with prucalopride 2 mg once daily compared with placebo during Weeks 2 through 12 (*p* values were not provided) [101].

#### Neuromodulators

Neuromodulators (e.g. antidepressants) have been shown to improve bloating in patients with IBS (including IBS-C and IBS-M), but data have generally been limited to small trials (<50 patients) [103,104]. In an RCT of patients with IBS-C (*n* = 44), the selective serotonin reuptake inhibitor fluoxetine 20 mg daily for 12 weeks significantly improved both constipation and bloating compared with placebo (*p* < 0.05) [103]. The largest RCT of tricyclic antidepressants (TCAs) in the management of IBS (ATLANTIS; Amitriptyline at Low-Dose and Titrated for Irritable Bowel Syndrome as Second-Line Treatment) included 463 patients with IBS (17% IBS-C, 41% IBS-M, 39% IBS-D, 3% unsubtyped) treated with amitriptyline (10–30 mg/day) or placebo for 6 months [105]. A significant decrease was observed for amitriptyline versus placebo in the IBS severity scoring system score and adequate relief of IBS symptoms (both *p* = 0.008). However, effects on abdominal distension were not statistically significant, and outcomes specific to bloating were not reported. Because the anticholinergic properties of tertiary amine TCAs such as amitriptyline and imipramine may worsen constipation, it has been proposed that neuromodulators with a lower potential to reduce intestinal motility, such as secondary amine TCAs (e.g. desipramine, nortriptyline) or serotonin-norepinephrine reuptake inhibitors (e.g. duloxetine, venlafaxine) may be more appropriate for patients with IBS-C, although supporting data remain limited [106,107].

##### Nonpharmacologic mechanical stimulation and neural modulation

Data are limited on the efficacy of mechanical and neuronal stimulation modalities for treating constipation with bloating. Direct mechanical stimulation of the colon using a vibrating capsule, indicated for the treatment of CIC in adults, did not improve mean bloating scores from baseline at Week 8 versus a placebo capsule (−0.3 vs −0.2; *p* = 0.55) in a phase 3 randomized trial of 312 adults with CIC (1 capsule/day, 5 days/week, for 8 weeks) [108]. Investigational therapies, including transabdominal electrical stimulation (randomized, double-blind, sham-controlled phase 3 trial) and sacral neuromodulation (randomized, open-label trial versus conservative treatment) have been evaluated in patients with chronic constipation, but changes in the symptom of bloating were not reported [109,110]. A preliminary nonrandomized trial of 55 women with chronic constipation refractory to biofeedback reported that sacral nerve stimulation improved both constipation and abdominal bloating [111]; however, a meta-analysis of RCTs demonstrated only marginal relief of constipation [112].

##### Antibiotic treatment for IMO

For patients diagnosed with IMO, combination antibiotic regimens can be considered, although the evidence base is limited [19] and practices vary. In one small (*n* = 32) randomized, double-blind, placebo-controlled trial of patients with IBS-C who had a methane-positive breath test result (i.e. indicative of IMO), treatment with the nonsystemic antibiotic rifaximin 1650 mg plus neomycin 1000 mg for 2 weeks significantly improved the severity of constipation (*p* < 0.001) and bloating (*p* = 0.02) from baseline compared with treatment with neomycin (plus placebo) over the course of the 4-week study [63]. Of interest, 10 of 15 patients treated with rifaximin plus neomycin had methane levels ≤3 ppm posttreatment and significantly lower constipation severity compared with those who had methane levels >3 ppm at the last visit (*p* = 0.02).

Use of antibiotics may be limited by concerns regarding the risk of *Clostridioides difficile* colitis and antimicrobial resistance, although the risk may be reduced with targeted, short-term treatment regimens. Because neomycin has the potential to cause ototoxicity and nephrotoxicity, even with short-term oral dosing, patients should be monitored for proteinuria, elevations in serum creatinine levels, and decreases in auditory acuity, with immediate discontinuation of therapy if toxicity is suspected. For patients who are not candidates for antibiotic therapy, it has been suggested that a 2-week course of an elemental diet (typically consisting of free amino acids, medium-chain triglycerides, monosaccharides or easily digestible saccharide polymers, and required vitamins/minerals) may provide some symptom benefit [113,114].

#### Psychotherapeutics

Several psychotherapeutics have been investigated for the management of IBS, including CBT (e.g. in-person, teleconference, self-administered/minimal contact, and group CBT), contingency management, dynamic psychotherapy, gut-directed hypnotherapy, stress management, and face-to-face multicomponent psychological therapy [39]. A meta-analysis of psychological therapies for IBS (*n* = 41 randomized clinical trials; 4072 participants) concluded that while several therapies showed efficacy, CBT and gut-directed hypnotherapy had a greater evidence base and long-term improvement in IBS symptoms [39]. Individual symptom (e.g. bloating) data are limited [115,116] because studies of psychological therapies in IBS typically have focused on global, rather than distinct, symptom outcome measures [117–122]. Of interest, a 2025 pilot trial that assessed a 12-week, self-guided hypnotherapy program in 25 patients with nonorganic GI disorders (e.g. functional bloating/distention, IBS, or functional dyspepsia) with predominant chronic bloating reported that bloating scores were significantly reduced from baseline to end of treatment (*p* = 0.003) and that 69.6% of patients had a treatment response (≥30% reduction from baseline) for bloating [123]. Although the effects of hypnotherapy on bloating were promising, the findings were limited by the small sample size and lack of a control group [123]. Questions have been raised regarding the lack of oversight governing hypnotherapy practice and insufficient training of some practitioners delivering gut-directed hypnotherapy [124].

Biofeedback may be considered for treatment of constipation in patients with dyssynergic defecation; however, long-term data are limited [125,126]. In patients with PFD and constipation, biofeedback to teach relaxation of the pelvic floor and anal sphincter muscles increased bowel movement frequency and reduced straining and bloating frequency [75]. After five weekly sessions, patients with constipation due to PFD/dyssynergia (*n* = 34) had significantly greater improvements from baseline in stool frequency (*p* < 0.001) and bloating frequency (*p* < 0.01) per week at 1-, 6-, and 12-month follow-up compared with patients with constipation due to slow transit in the absence of dyssynergia (*n* = 12) [75]. Another study of 50 patients with constipation due to PFD (*n* = 36), slow transit (*n* = 8), or mixed etiology (*n* = 6) reported that biofeedback (electromyography- or manometry-based) 2 times weekly (total, five sessions) significantly reduced the percentage of patients with ‘difficult evacuation’ (i.e. constipation), from all patients at baseline to fewer than 35% at both 10 days and 1 year after completion of biofeedback sessions (*p* < 0.01 for both timepoints) [127]. Similarly, the percentage of patients with symptoms of ‘distension or bloating’ was significantly reduced, from 84% of patients at baseline to ≤30% after biofeedback (*p* < 0.05 for both timepoints). Furthermore, in a trial of 156 patients with a disorder of gut-brain interaction and severe bloating as a primary complaint, 105 (67.3%) patients did not respond to dietary advice, and 104 underwent a standardized balloon expulsion test [128]. Of the 67 (64.4%) who failed the balloon expulsion test, 65 underwent pelvic floor biofeedback therapy. After therapy, 53.8% of the 65 patients experienced fair improvement or major improvement/cure (i.e. responded [score, 3 or 4 on a 0- to 4-point scale]), and all responders had at least a 50% reduction in bloating intensity. Collectively, these findings support the potential utility of pelvic floor biofeedback in individuals with PFD experiencing constipation and/or bloating. However, the evidence is limited by relatively small sample sizes, lack of a control group, and short duration of follow-up. Outcomes may be further impacted by therapist skill and experience, as well as variability in the specific training techniques implemented [75]. For patients with PFD who do not respond to or are not candidates for biofeedback, sacral neuromodulation, discussed previously, has been suggested as a safe alternative [129,130].

## Discussion

Although constipation and bloating are both common GI symptoms that present together, bloating is a relatively nonspecific symptom with a complex pathophysiology. For example, while bloating in the presence of constipation can occur as the result of luminal distension of the intestines by excess stool and gas, there can be other factors, such as visceral hypersensitivity and alterations in the gut microbiome, as well as dietary and psychological influences, which can affect symptom presentation in the individual patient. Thus, while simply treating constipation *via* the use of medications (such as a secretagogue, chloride channel activator, or sodium/hydrogen exchanger isoform 3 inhibitor) or pelvic floor physical therapy (with biofeedback therapy in the setting of PFD) can improve bloating, unfortunately, effective treatment of constipation does not resolve bloating for all patients. In these cases, there is likely another underlying factor(s) contributing to the manifestation of bloating symptoms. Therefore, there is a need for additional research aimed at understanding the physiology of bloating to improve treatment options for patients with and without constipation.

Because bloating is common in patients with disorders of gut-brain interaction, such as IBS and functional dyspepsia, it is reasonable to hypothesize that interventions focused on modulating the brain-gut axis, such as neuromodulators and psychological therapies (e.g. CBT and hypnotherapy), as well as other novel techniques (e.g. virtual reality) are treatment strategies ripe for further exploration with regard to the treatment of bloating. Additionally, the identification of other pathophysiological causes of bloating, such as altered intestinal barrier function and colonic dysbiosis, may help to identify new treatment targets for bloating. Furthermore, while SIBO and increasingly IMO are being considered in the diagnostic strategy for patients with bloating, the true prevalence among patients with bloating as a predominant symptom is unknown. Substantial debate surrounds the ideal testing parameters (i.e. breath testing) for accurate diagnosis of SIBO and IMO. Additional research is needed to better understand the true prevalence of SIBO and IMO in patients with bloating as a primary symptom, as well as to clarify ideal testing strategies to allow for more educated and judicious use of antibiotic treatment in clinical practice. Finally, while patients often attribute symptoms of bloating to diet, many fail to achieve adequate symptom relief with various diet elimination trials. A low FODMAP diet currently has the best evidence for use as a diet strategy to manage symptoms of IBS, including bloating, but it does not work for all patients. For example, data are lacking regarding the utility of the low FODMAP diet to manage specific symptoms of IBS-C. Based on available evidence, no diet can be universally recommended for the treatment of bloating as a primary symptom. Further research assessing diet to treat bloating, particularly in the setting of constipation, is needed. In an era when personalized medicine is more commonly emphasized, understanding how and why bloating presents in an individual patient (whether with constipation or other concomitant GI and non-GI symptoms) will be paramount to improve treatment outcomes for this common, frequently vexing symptom.

## Conclusions

Constipation and bloating are common GI symptoms that frequently occur together. While the etiology for constipation and bloating is diverse, specific GI causes that should be considered in the evaluation of bloating and constipation include CIC, IBS-C, IBS-M, PFD, and IMO. In cases for which food intolerance is suspected, a 2-week trial of dietary restriction to assess response (e.g. low FODMAP diet) can be considered, ideally with the assistance of a qualified dietician. Furthermore, soluble fiber and polyethylene glycol are safe and reasonable first-line treatment considerations. Among pharmacologic options, secretagogues indicated for the treatment of IBS-C and CIC have demonstrated significant improvements in constipation and bloating symptoms in robust RCTs. Other treatments that may have a role for patients with constipation and bloating include antibiotics (bacterial overgrowth), psychotherapeutic approaches (IBS-C), and pelvic floor physical therapy with biofeedback (PFD); however, data are relatively limited, and more well-designed RCTs are needed. An individualized approach, based on available evidence, is advised to best address the common and frequently bothersome symptoms of constipation and bloating.

Looking ahead, additional research is needed to better understand the prevalence of SIBO and IMO in patients with bloating, as these remain popular and oft-debated topics in the field of gastroenterology and the true prevalence of these conditions remains unclear. Further studies are needed to establish the most effective treatment strategies for microbial overgrowth conditions, such as SIBO and IMO. In addition, clinical trials evaluating new therapeutic approaches for bloating – particularly dietary interventions, neuromodulators, and psychologically based therapies – would be valuable, as these modalities are likely to play an increasingly prominent role in managing bloating and constipation in the future.

## Data Availability

Data sharing is not applicable to this article, as no data were created or analyzed in this research.
